# Supplementary material for: Receptor tyrosine kinases CAD96CA and FGFR1 function as the cell membrane receptors of insect juvenile hormone
Source: eLife. 2025 Mar 14;13:RP97189. doi: 10.7554/eLife.97189 (PMC11908783; doi:10.7554/eLife.97189)
Supplement: Figure 2—source data 2. [file elife-97189-fig2-data2.pdf]

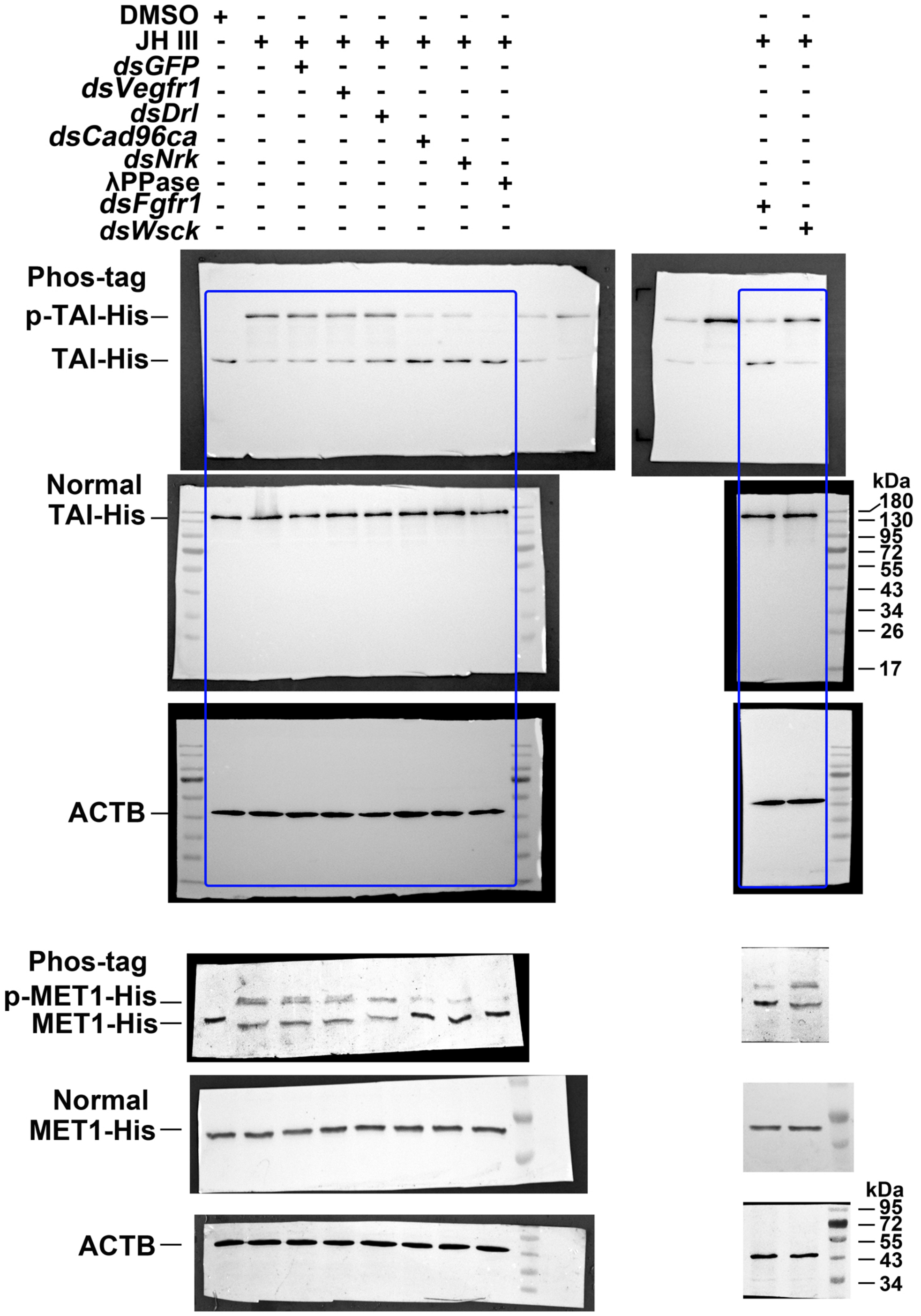

Figure 2C, Source Data 2. Original western blot images corresponding to Figure 2C. The areas enclosed by the boxs represent the content shown in Figure 2C.
