# Supplementary figures and images for: Receptor tyrosine kinases CAD96CA and FGFR1 function as the cell membrane receptors of insect juvenile hormone
Source: eLife. 2025 Mar 14;13:RP97189. doi: 10.7554/eLife.97189 (PMC11908783; doi:10.7554/eLife.97189)

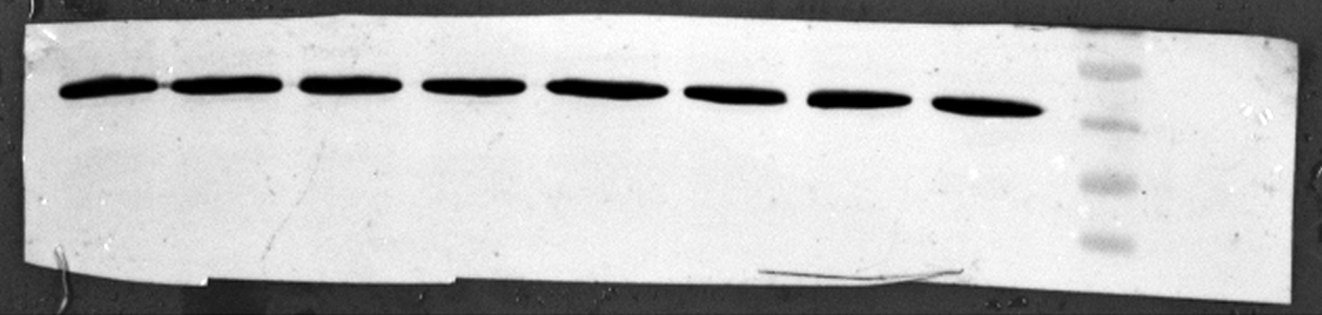

Supplement: Figure 2—source data 3. [file elife-97189-fig2-data3.zip › Figure 2-Source Data 3/MET1-His ACTB-1.tif]

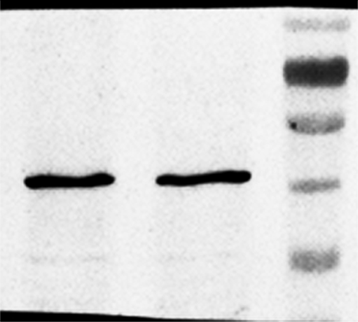

Supplement: Figure 2—source data 3. [file elife-97189-fig2-data3.zip › Figure 2-Source Data 3/MET1-His ACTB-2.tif]

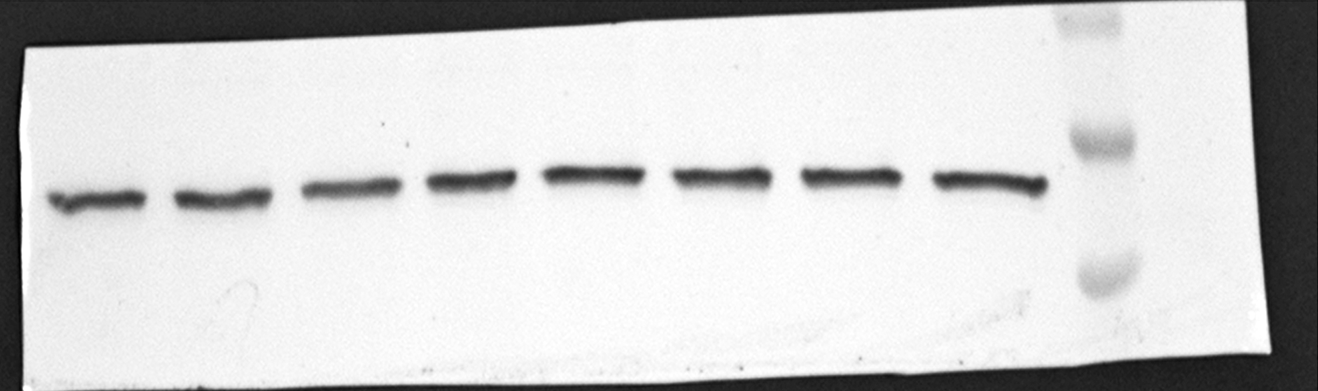

Supplement: Figure 2—source data 3. [file elife-97189-fig2-data3.zip › Figure 2-Source Data 3/Normal MET1-His-1.tif]

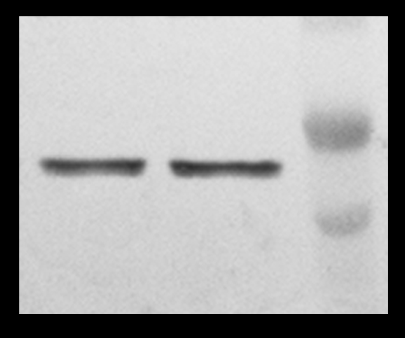

Supplement: Figure 2—source data 3. [file elife-97189-fig2-data3.zip › Figure 2-Source Data 3/Normal MET1-His-2.tif]

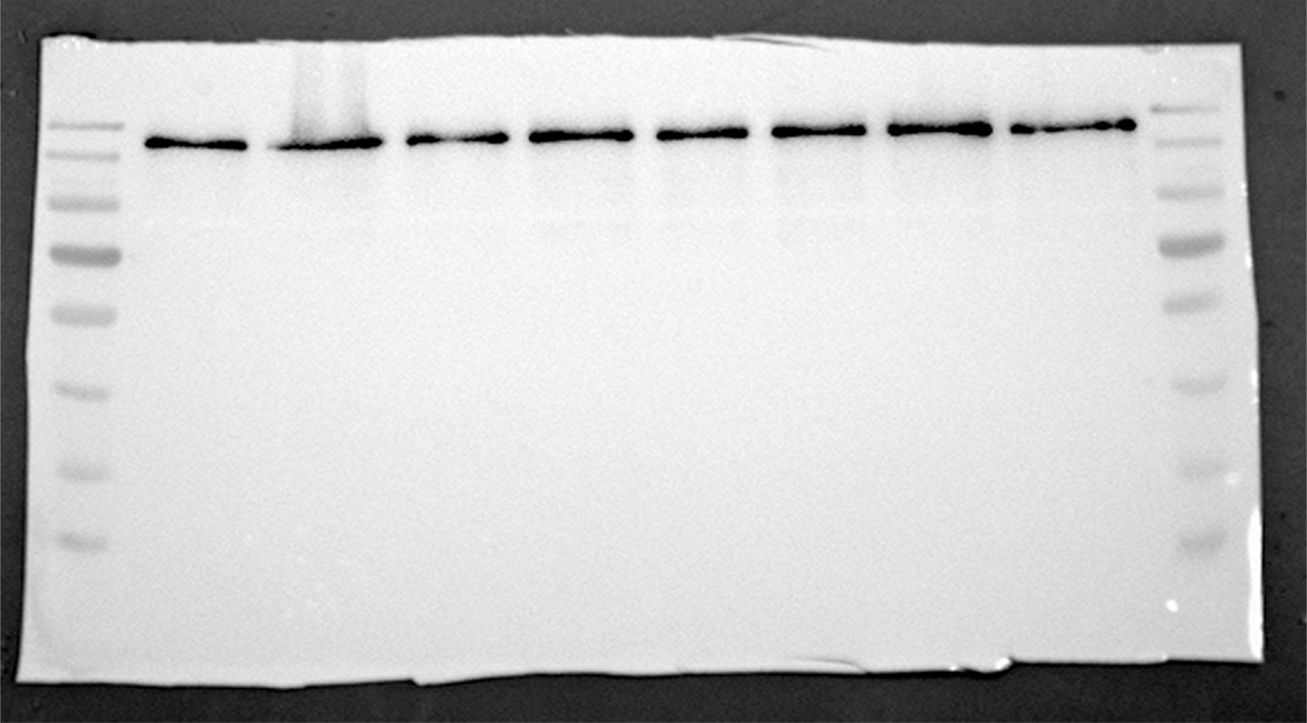

Supplement: Figure 2—source data 3. [file elife-97189-fig2-data3.zip › Figure 2-Source Data 3/Normal TAI-His-1.tif]

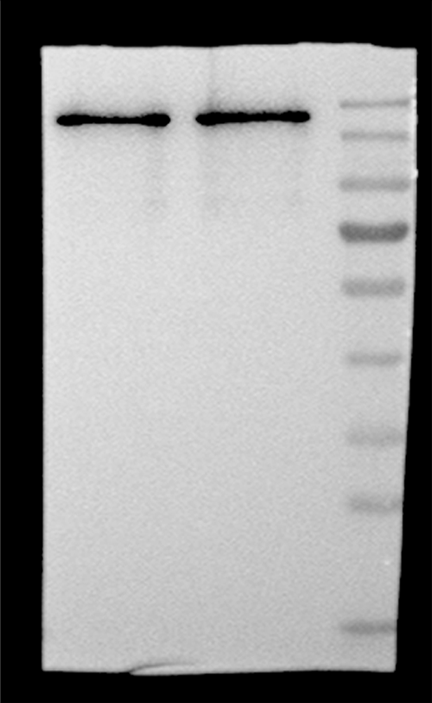

Supplement: Figure 2—source data 3. [file elife-97189-fig2-data3.zip › Figure 2-Source Data 3/Normal TAI-His-2.tif]

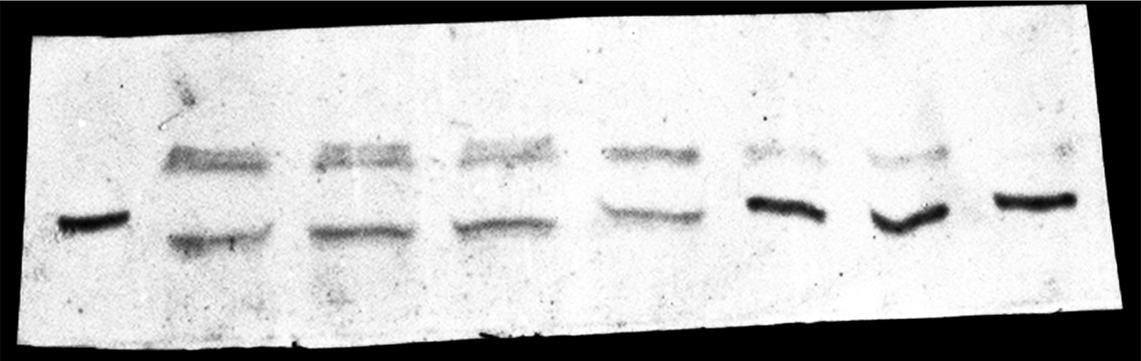

Supplement: Figure 2—source data 3. [file elife-97189-fig2-data3.zip › Figure 2-Source Data 3/Phos-tag MET1-His-1.tif]

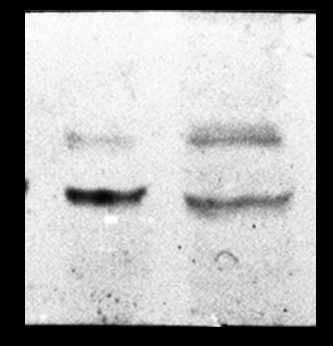

Supplement: Figure 2—source data 3. [file elife-97189-fig2-data3.zip › Figure 2-Source Data 3/Phos-tag MET1-His-2.tif]

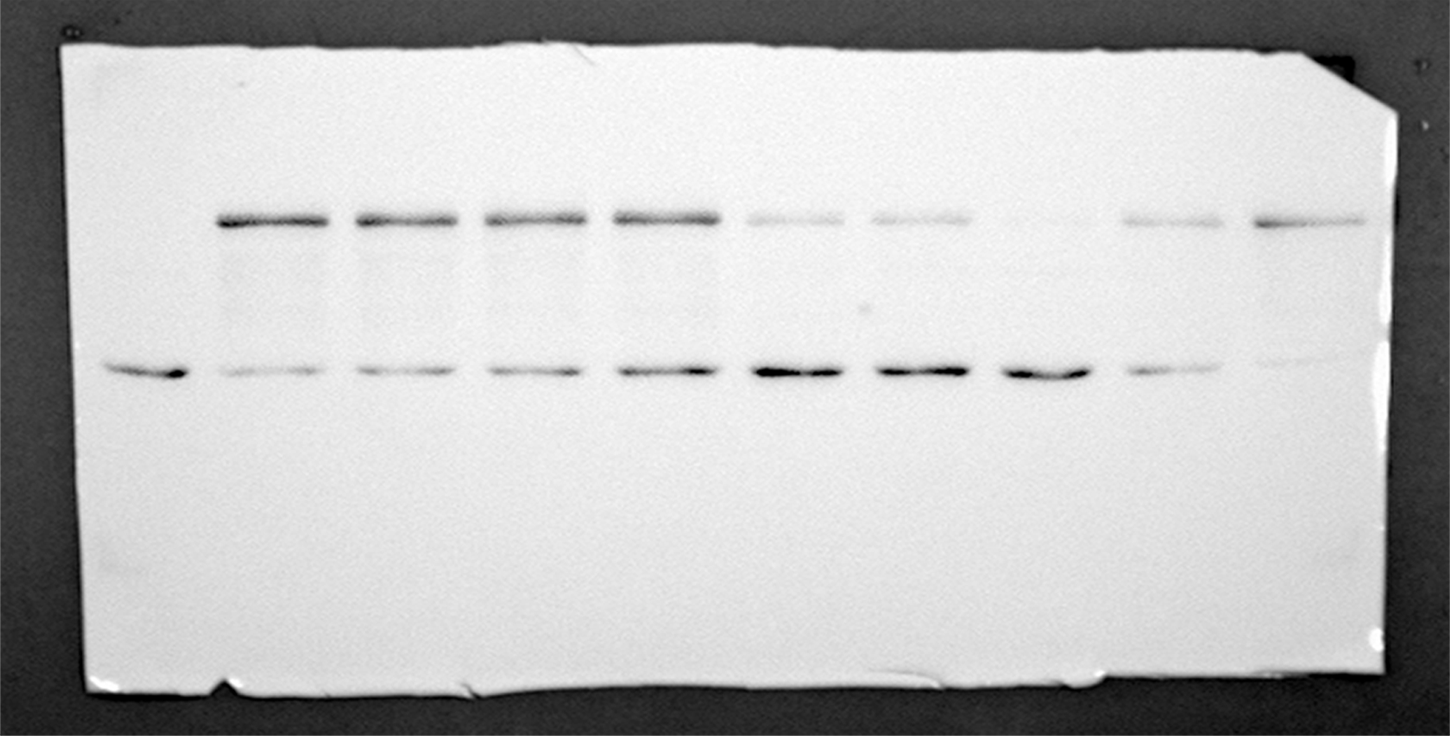

Supplement: Figure 2—source data 3. [file elife-97189-fig2-data3.zip › Figure 2-Source Data 3/Phos-tag TAI-His-1.tif]

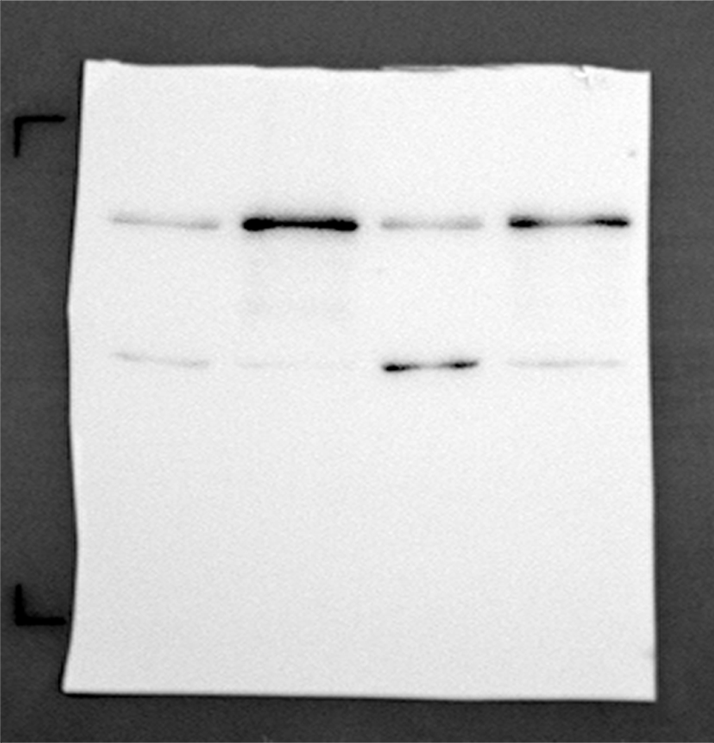

Supplement: Figure 2—source data 3. [file elife-97189-fig2-data3.zip › Figure 2-Source Data 3/Phos-tag TAI-His-2.tif]

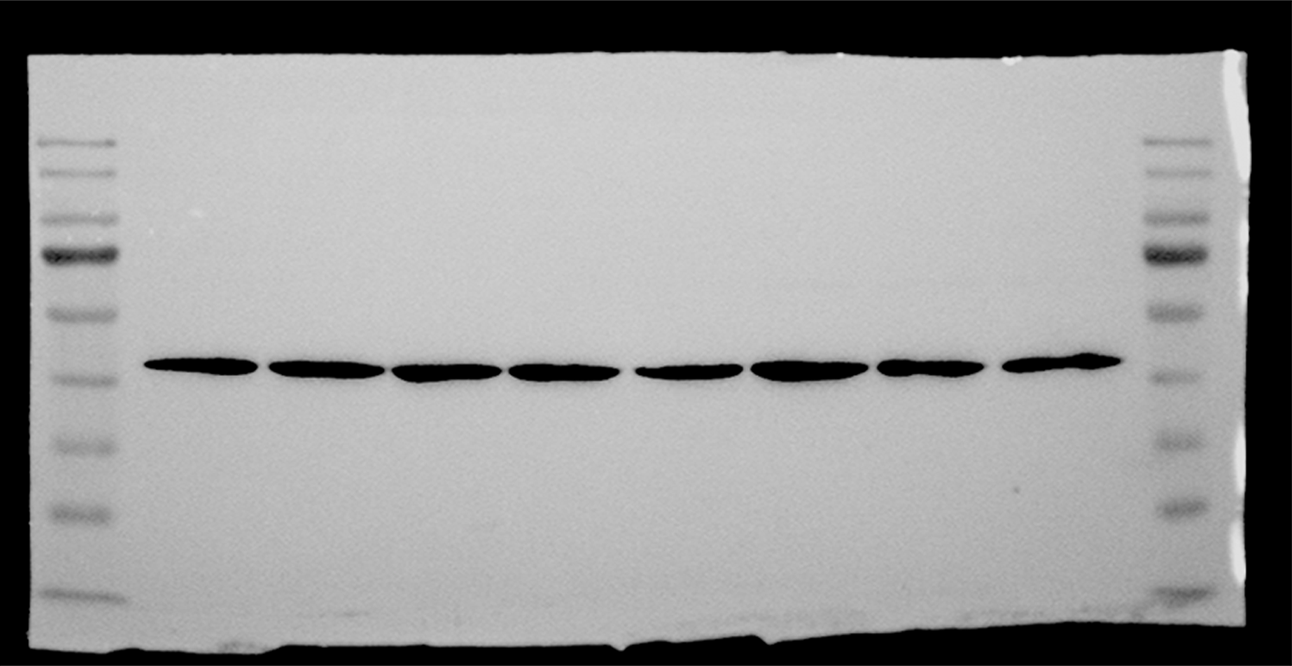

Supplement: Figure 2—source data 3. [file elife-97189-fig2-data3.zip › Figure 2-Source Data 3/TAI-His ACTB-1.tif]

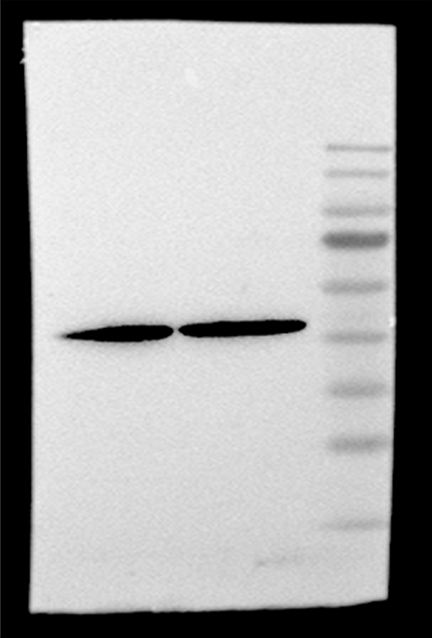

Supplement: Figure 2—source data 3. [file elife-97189-fig2-data3.zip › Figure 2-Source Data 3/TAI-His ACTB-2.tif]

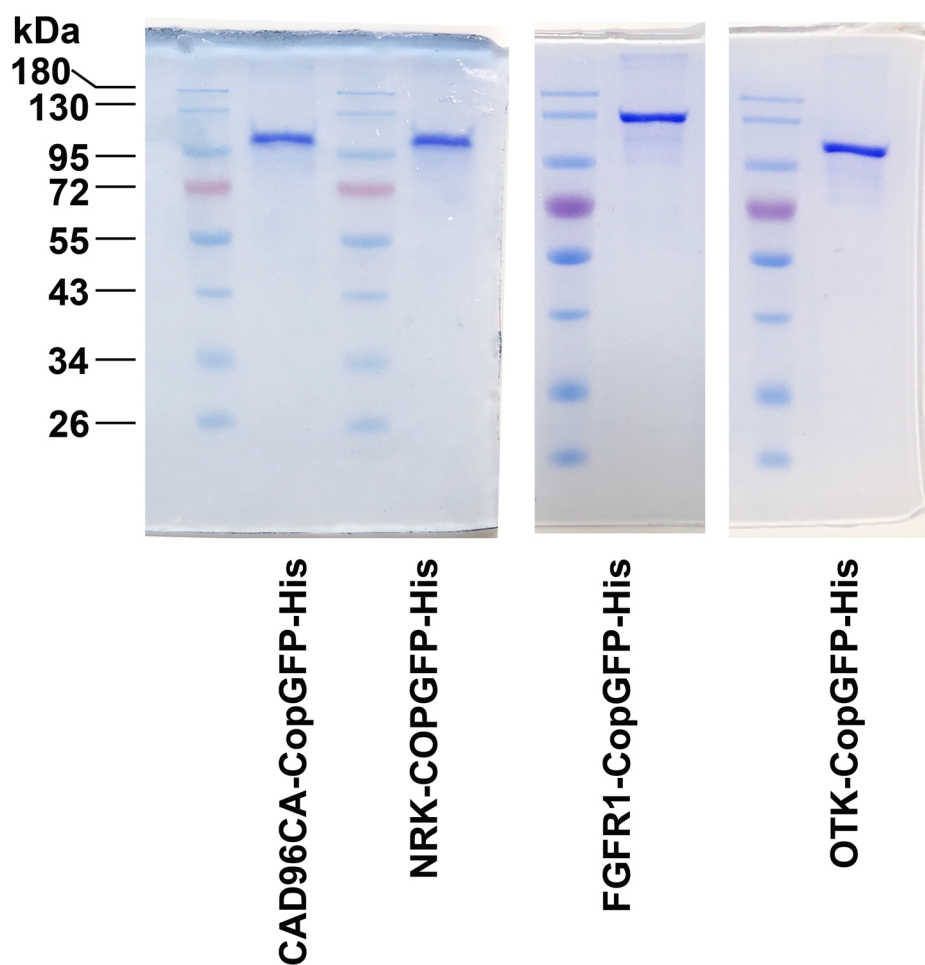

Figure 3B, Source Data 2. Original gel images corresponding to Figure 3B.

Supplement: Figure 3—source data 2. [file elife-97189-fig3-data2.pdf]

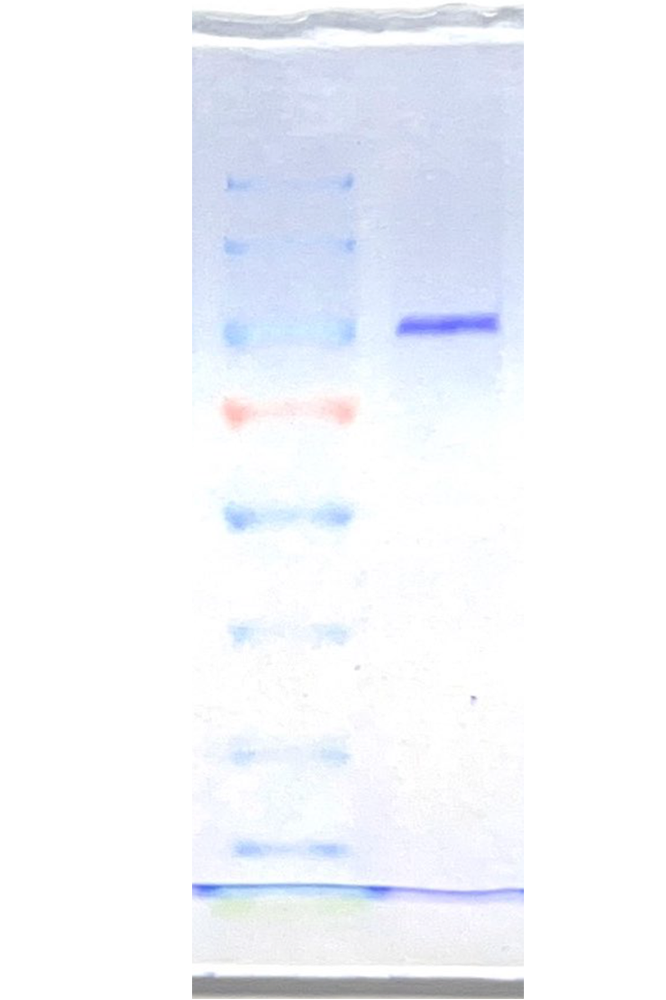

Supplement: Figure 3—figure supplement 1—source data 3. [file elife-97189-fig3-figsupp1-data3.zip › Figure 3–figure supplement 1-Source Data 3/CAD96CA-M1-CopGFP-His.tif]

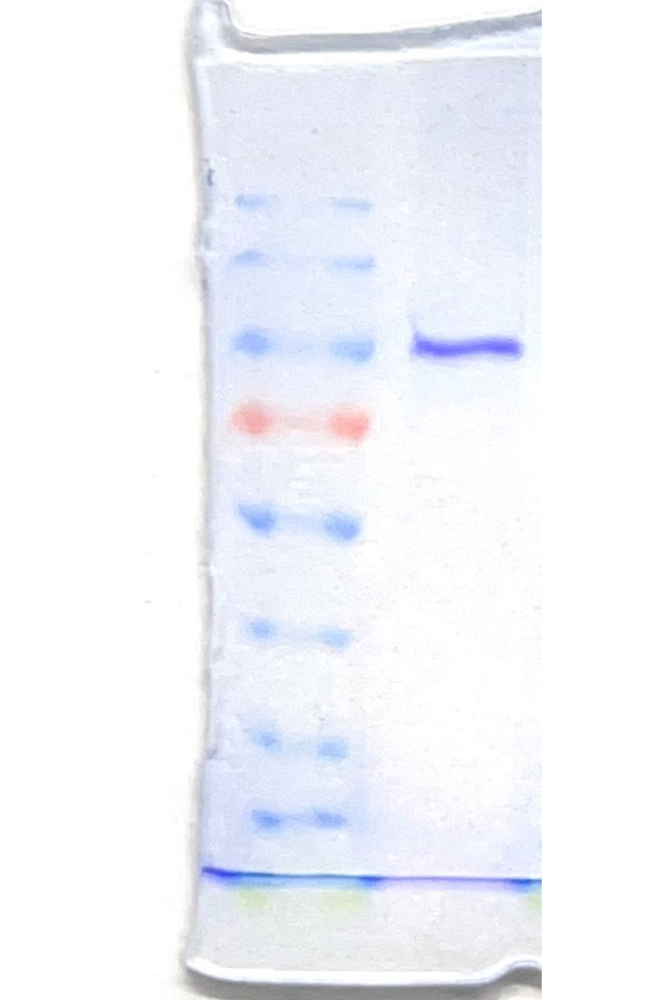

Supplement: Figure 3—figure supplement 1—source data 3. [file elife-97189-fig3-figsupp1-data3.zip › Figure 3–figure supplement 1-Source Data 3/CAD96CA-M2-CopGFP-His.tif]

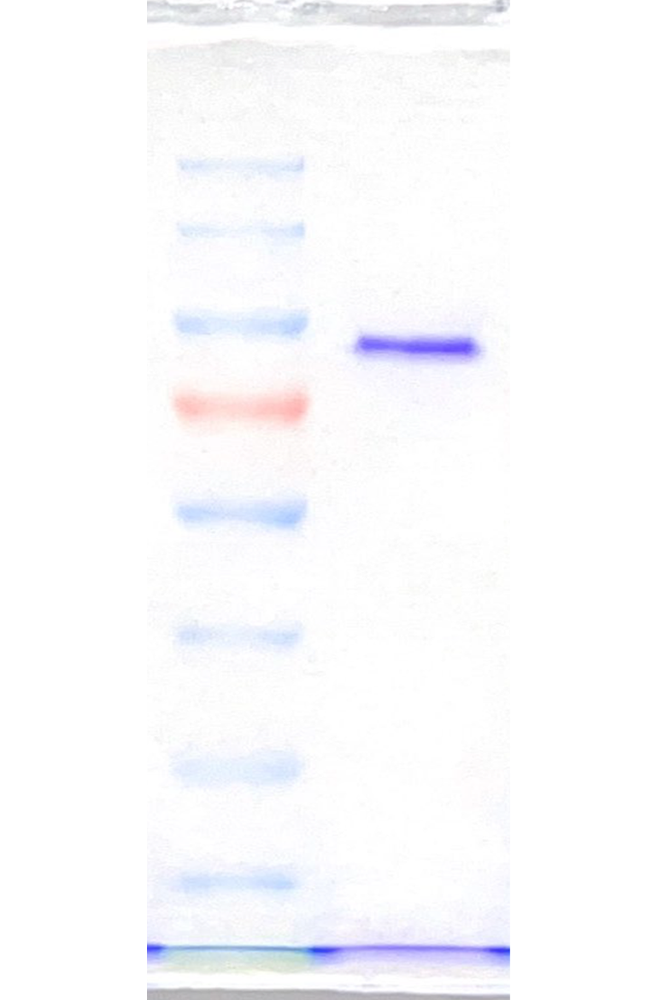

Supplement: Figure 3—figure supplement 1—source data 3. [file elife-97189-fig3-figsupp1-data3.zip › Figure 3–figure supplement 1-Source Data 3/CAD96CA-M3-CopGFP-His.tif]

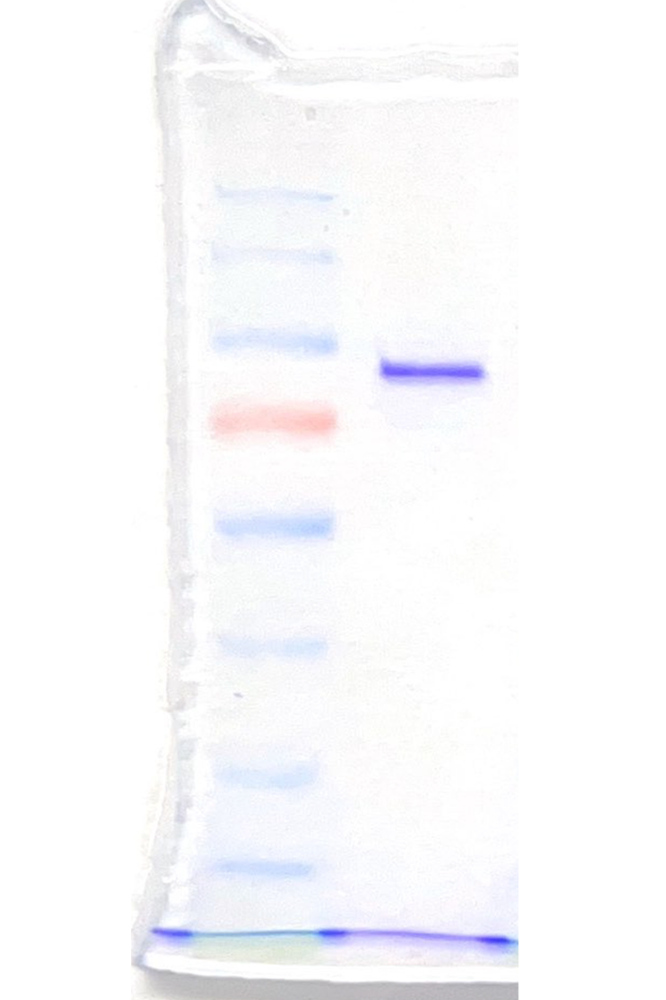

Supplement: Figure 3—figure supplement 1—source data 3. [file elife-97189-fig3-figsupp1-data3.zip › Figure 3–figure supplement 1-Source Data 3/CAD96CA-M4-CopGFP-His.tif]

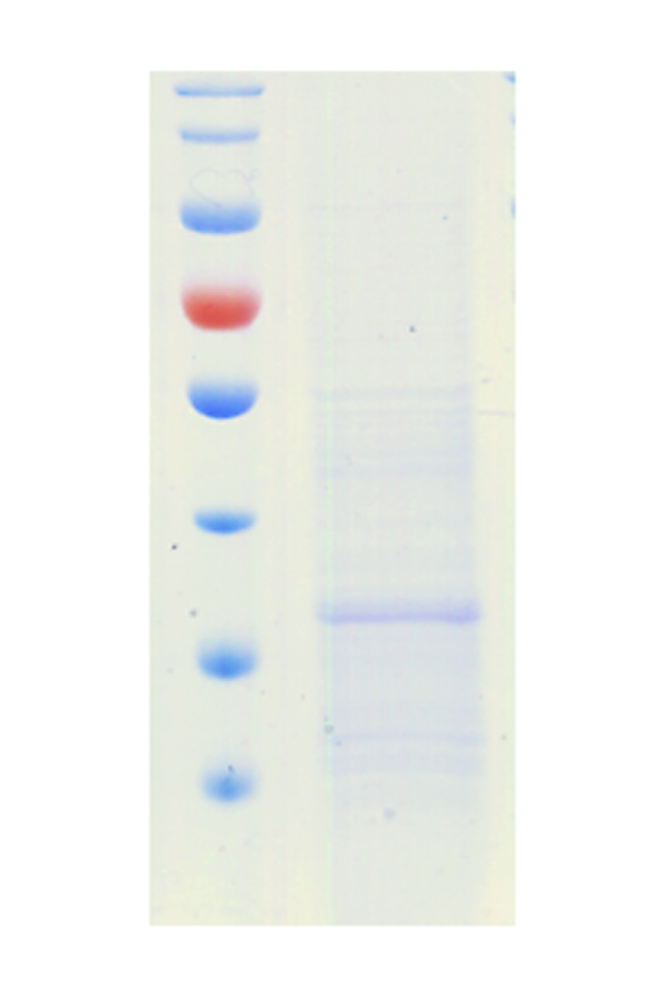

Supplement: Figure 3—figure supplement 1—source data 3. [file elife-97189-fig3-figsupp1-data3.zip › Figure 3–figure supplement 1-Source Data 3/CopGFP-His.tif]

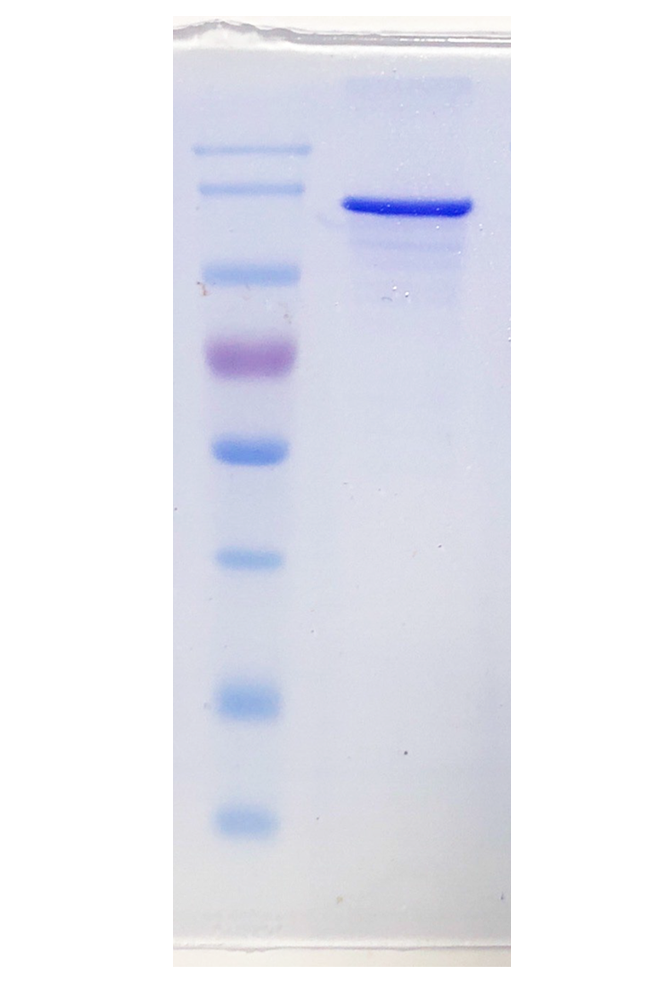

Supplement: Figure 3—figure supplement 1—source data 3. [file elife-97189-fig3-figsupp1-data3.zip › Figure 3–figure supplement 1-Source Data 3/FGFR1-M1-CopGFP-His.tif]

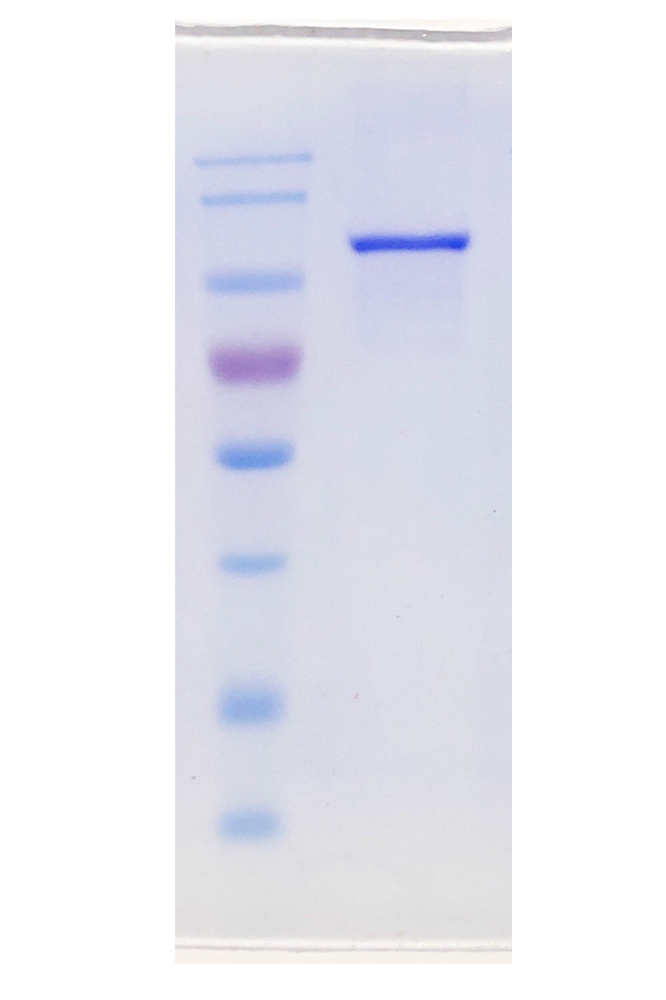

Supplement: Figure 3—figure supplement 1—source data 3. [file elife-97189-fig3-figsupp1-data3.zip › Figure 3–figure supplement 1-Source Data 3/FGFR1-M2-CopGFP-His.tif]

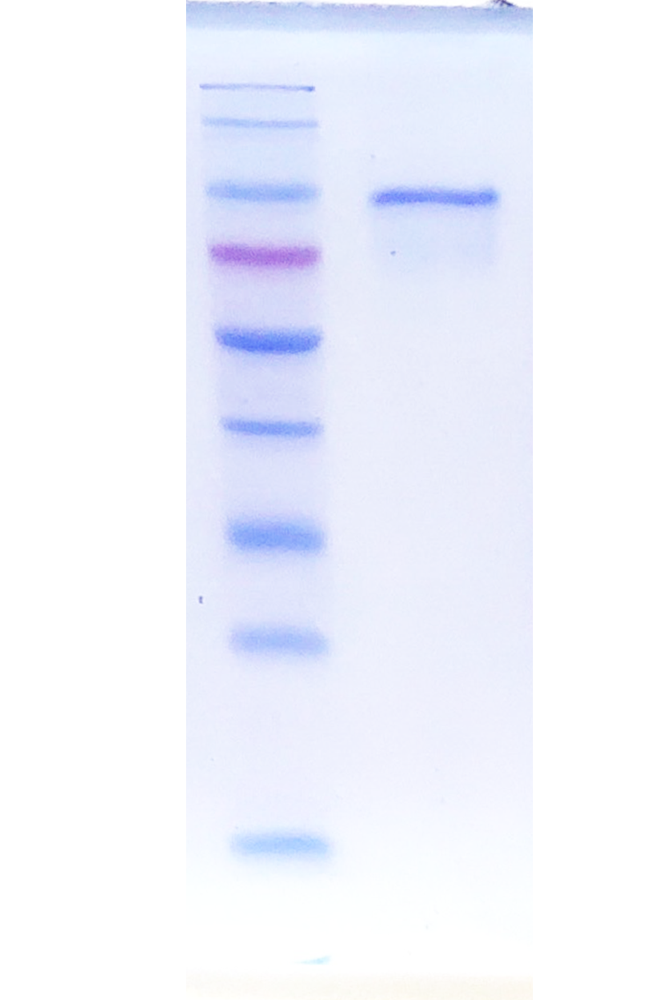

Supplement: Figure 3—figure supplement 1—source data 3. [file elife-97189-fig3-figsupp1-data3.zip › Figure 3–figure supplement 1-Source Data 3/FGFR1-M3-CopGFP-His.tif]

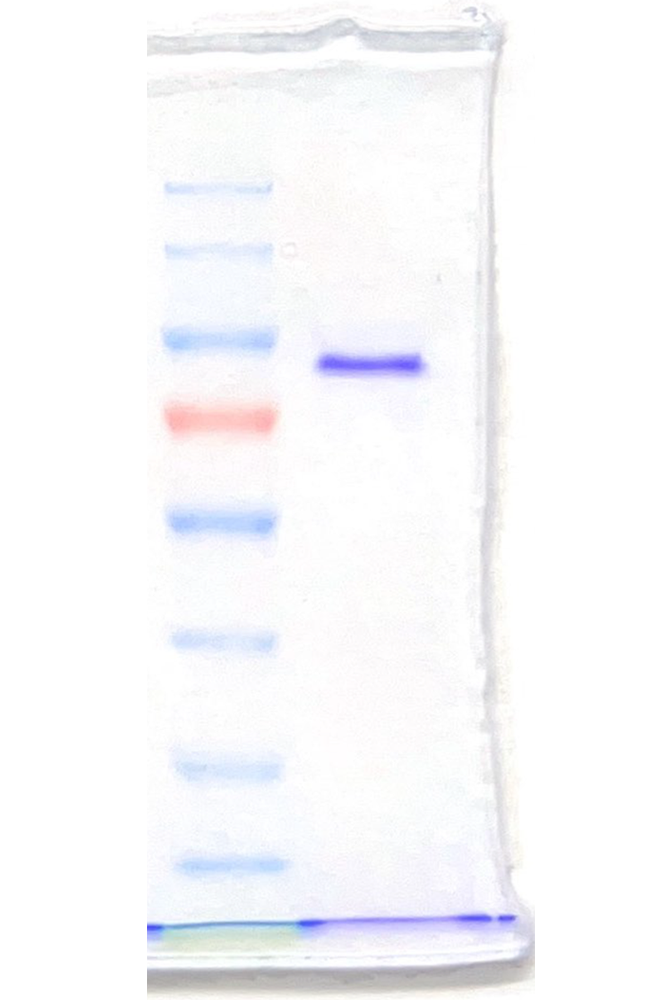

Supplement: Figure 3—figure supplement 1—source data 3. [file elife-97189-fig3-figsupp1-data3.zip › Figure 3–figure supplement 1-Source Data 3/FGFR1-M4-CopGFP-His.tif]

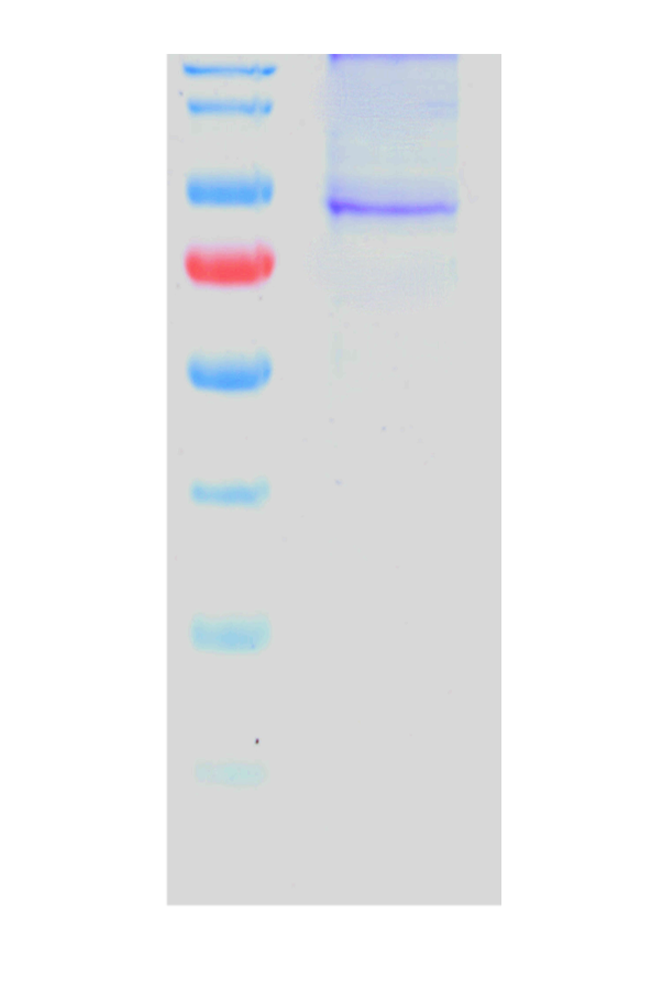

Supplement: Figure 3—figure supplement 1—source data 3. [file elife-97189-fig3-figsupp1-data3.zip › Figure 3–figure supplement 1-Source Data 3/MET1-CopGFP-His.tif]

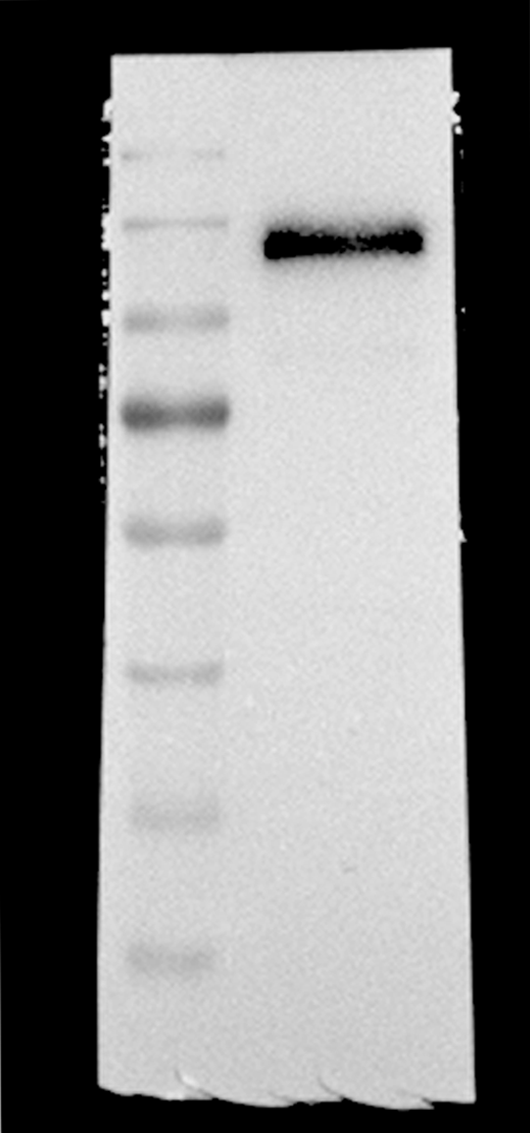

Supplement: Figure 6—figure supplement 2—source data 2. [file elife-97189-fig6-figsupp2-data2.zip › Figure 6–figure supplement 2-Source Data 2/CAD96CA-GFP-.tif]

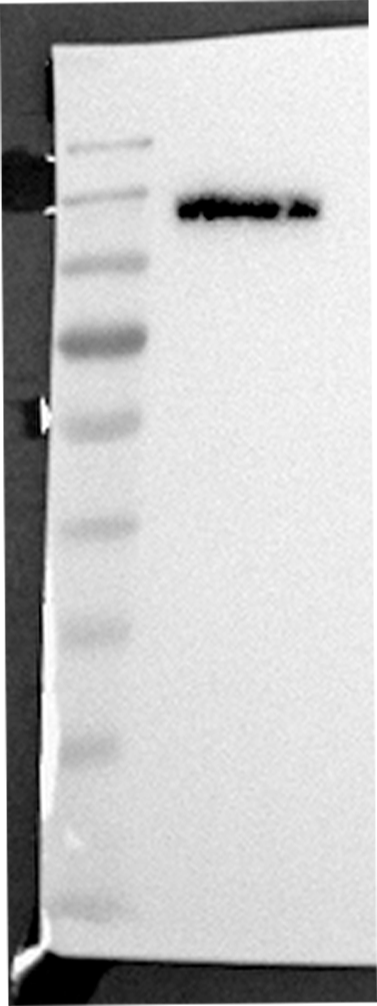

Supplement: Figure 6—figure supplement 2—source data 2. [file elife-97189-fig6-figsupp2-data2.zip › Figure 6–figure supplement 2-Source Data 2/CAD96CA-GFP.tif]

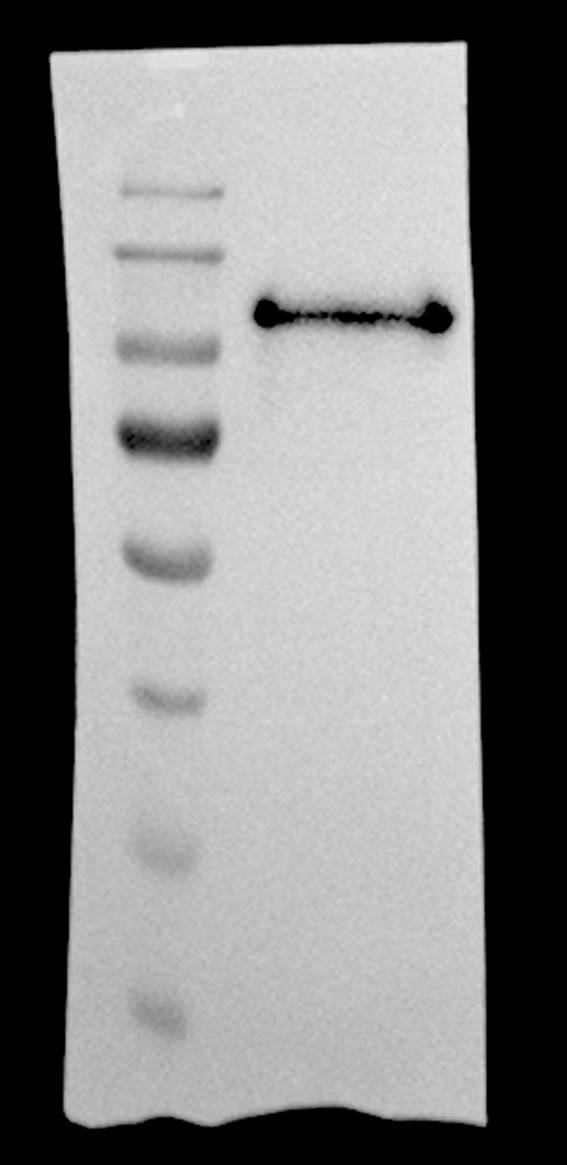

Supplement: Figure 6—figure supplement 2—source data 2. [file elife-97189-fig6-figsupp2-data2.zip › Figure 6–figure supplement 2-Source Data 2/CAD96CA-M1-GFP.tif]

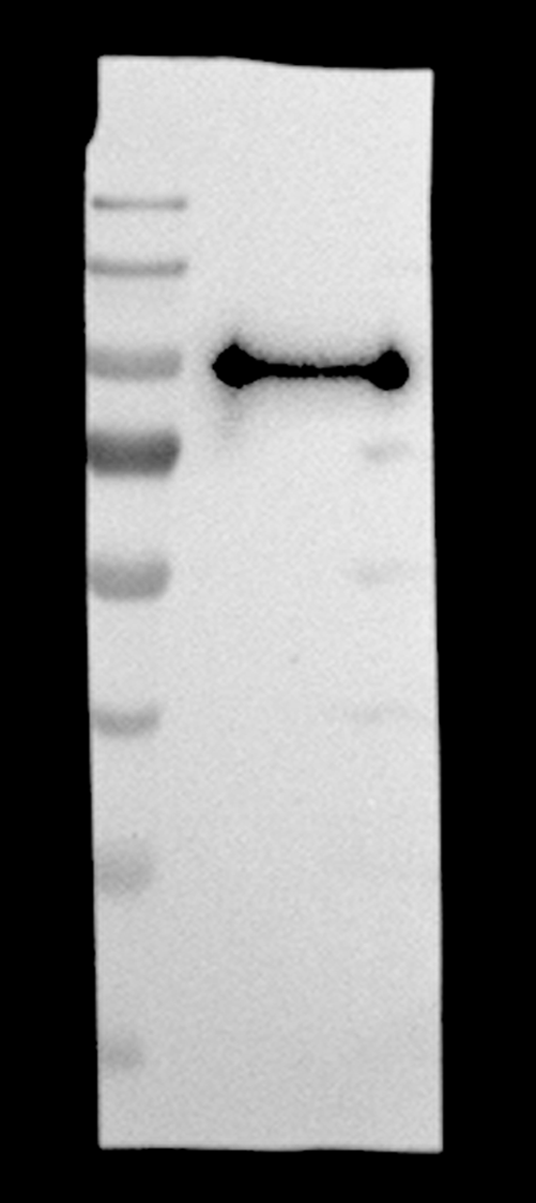

Supplement: Figure 6—figure supplement 2—source data 2. [file elife-97189-fig6-figsupp2-data2.zip › Figure 6–figure supplement 2-Source Data 2/CAD96CA-M2-GFP.tif]

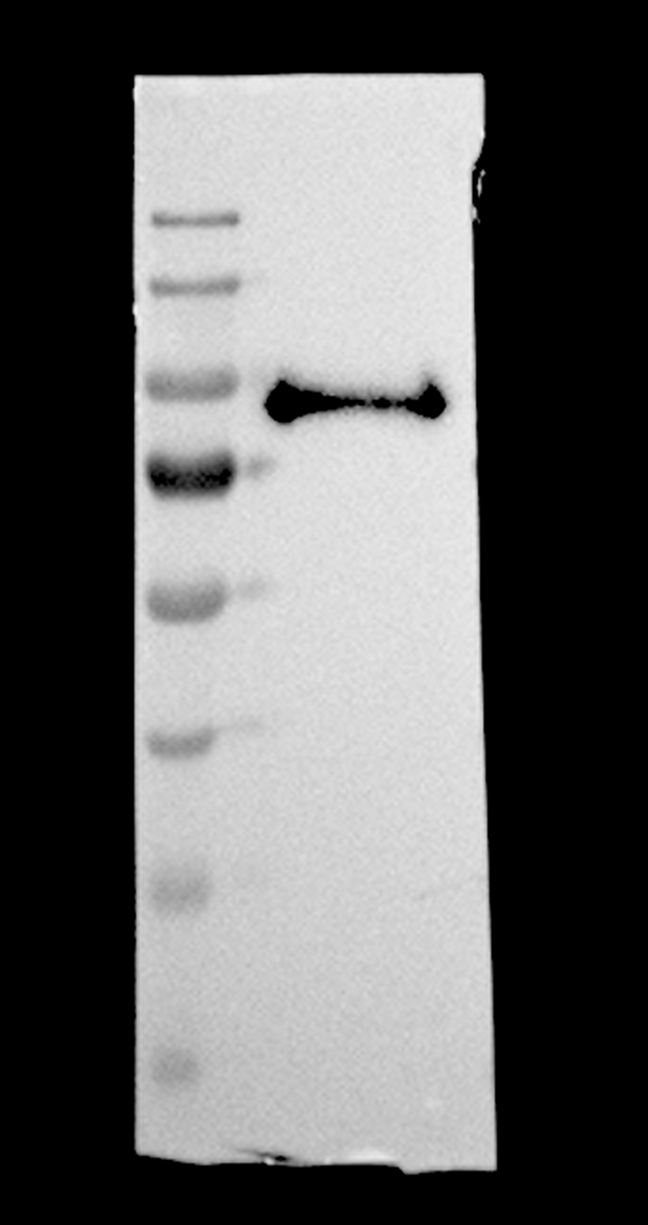

Supplement: Figure 6—figure supplement 2—source data 2. [file elife-97189-fig6-figsupp2-data2.zip › Figure 6–figure supplement 2-Source Data 2/CAD96CA-M3-GFP.tif]

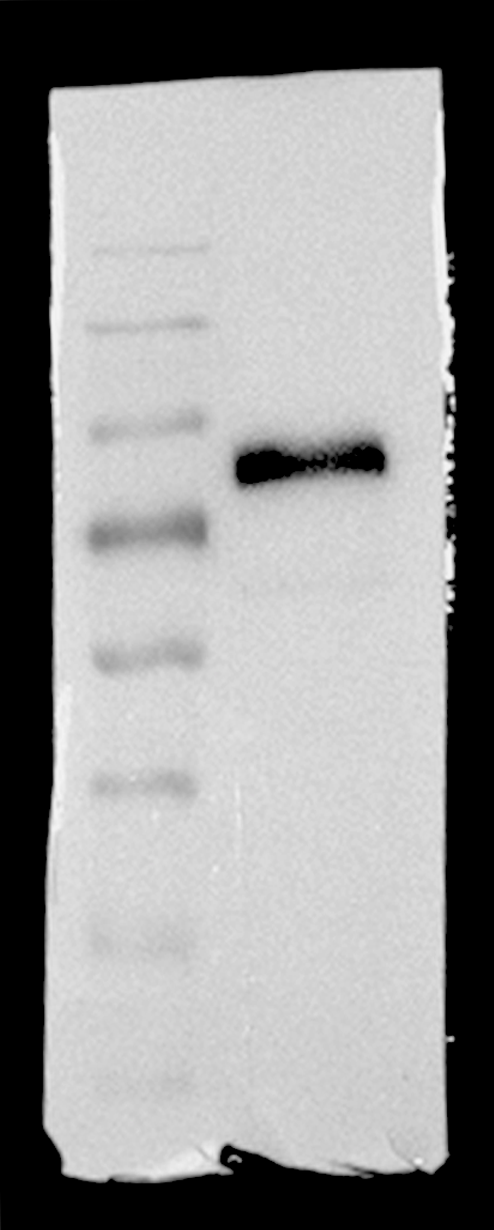

Supplement: Figure 6—figure supplement 2—source data 2. [file elife-97189-fig6-figsupp2-data2.zip › Figure 6–figure supplement 2-Source Data 2/CAD96CA-M4-GFP.tif]

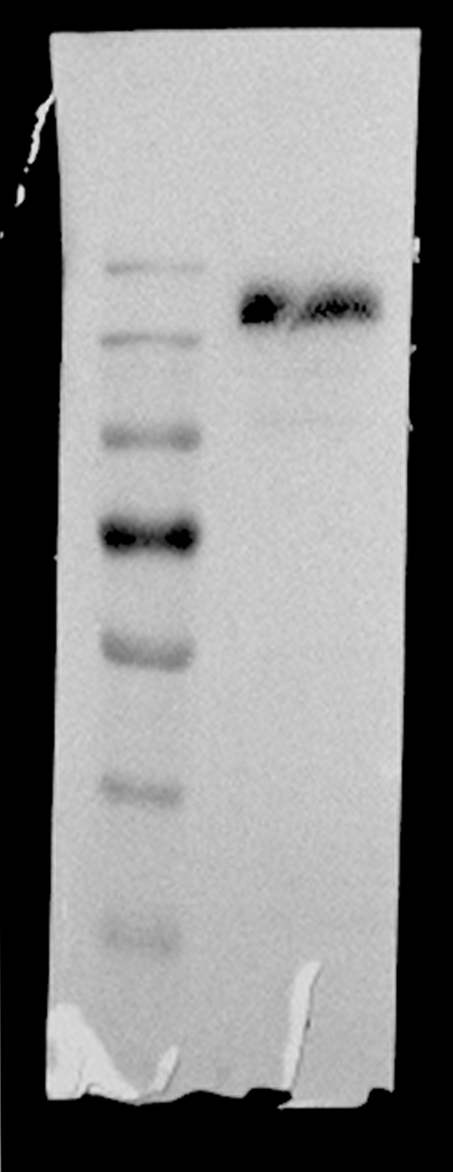

Supplement: Figure 6—figure supplement 2—source data 2. [file elife-97189-fig6-figsupp2-data2.zip › Figure 6–figure supplement 2-Source Data 2/FGFR1-GFP-.tif]

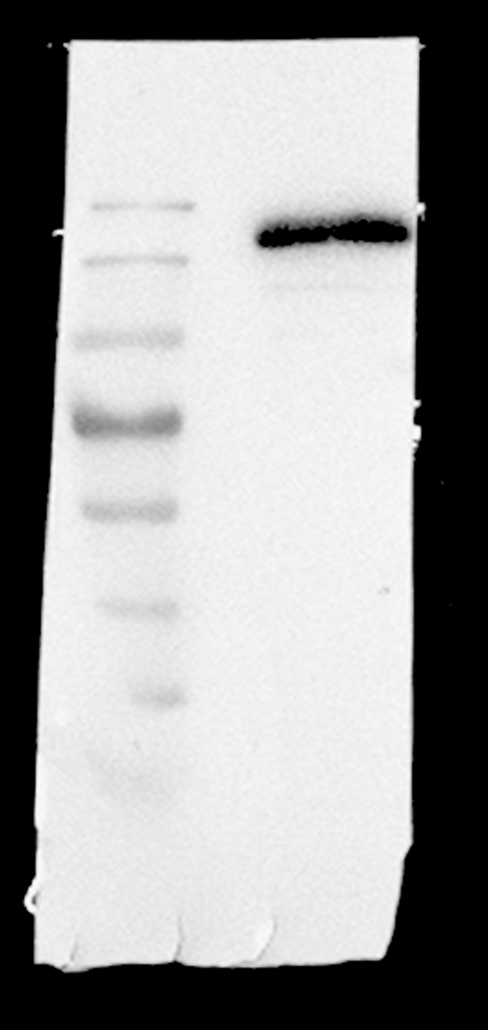

Supplement: Figure 6—figure supplement 2—source data 2. [file elife-97189-fig6-figsupp2-data2.zip › Figure 6–figure supplement 2-Source Data 2/FGFR1-GFP.tif]

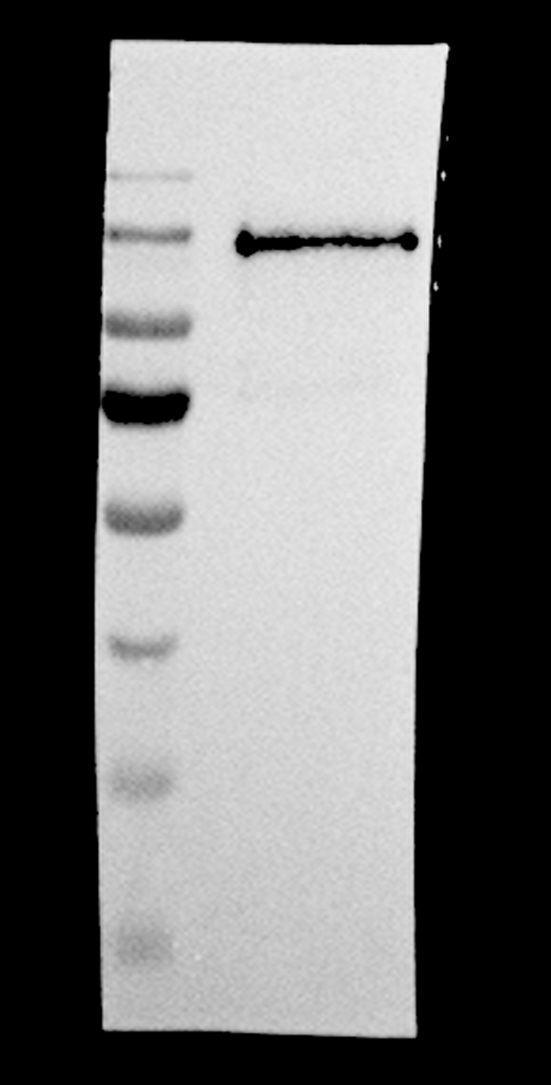

Supplement: Figure 6—figure supplement 2—source data 2. [file elife-97189-fig6-figsupp2-data2.zip › Figure 6–figure supplement 2-Source Data 2/FGFR1-M1-GFP.tif]

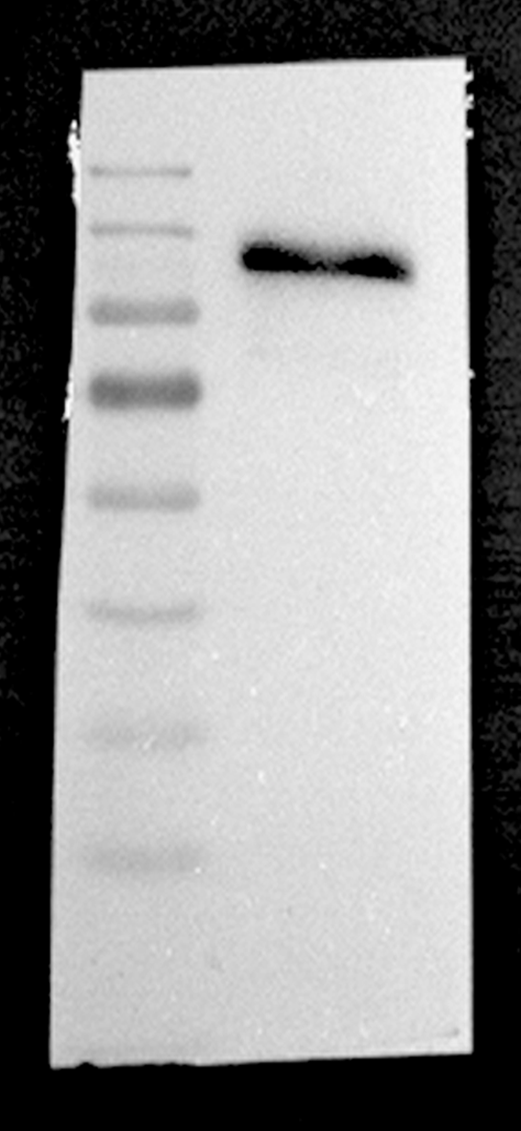

Supplement: Figure 6—figure supplement 2—source data 2. [file elife-97189-fig6-figsupp2-data2.zip › Figure 6–figure supplement 2-Source Data 2/FGFR1-M2-GFP.tif]

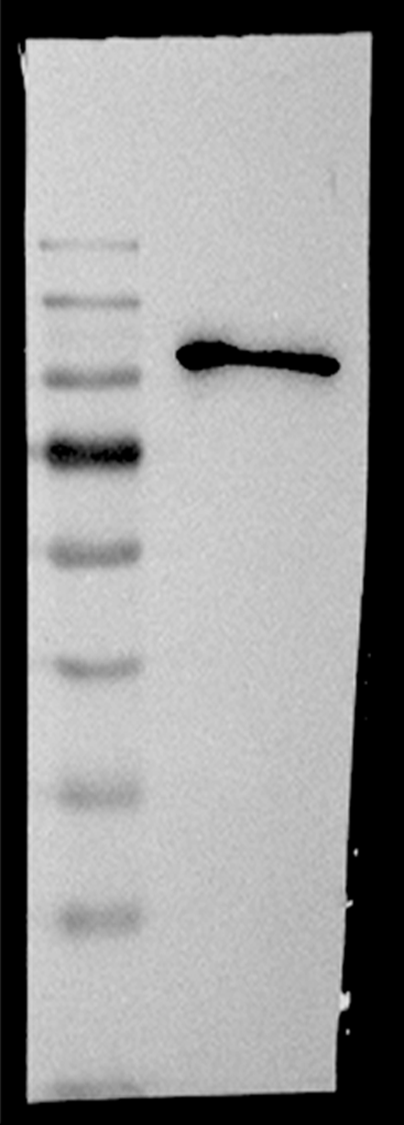

Supplement: Figure 6—figure supplement 2—source data 2. [file elife-97189-fig6-figsupp2-data2.zip › Figure 6–figure supplement 2-Source Data 2/FGFR1-M3-GFP.tif]

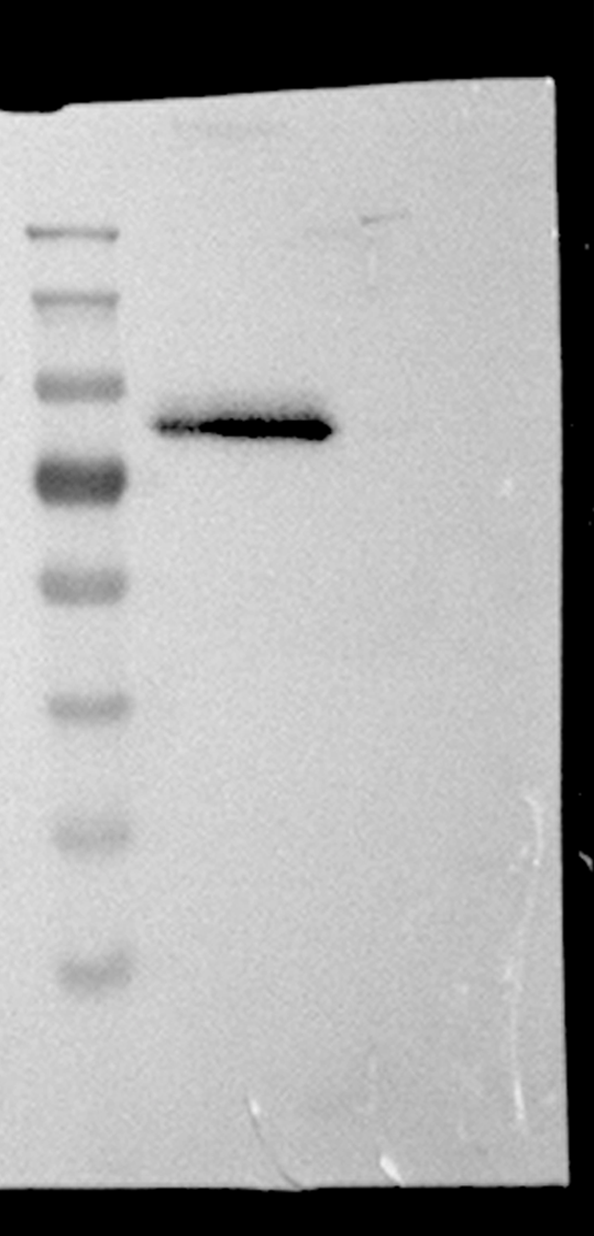

Supplement: Figure 6—figure supplement 2—source data 2. [file elife-97189-fig6-figsupp2-data2.zip › Figure 6–figure supplement 2-Source Data 2/FGFR1-M4-GFP.tif]

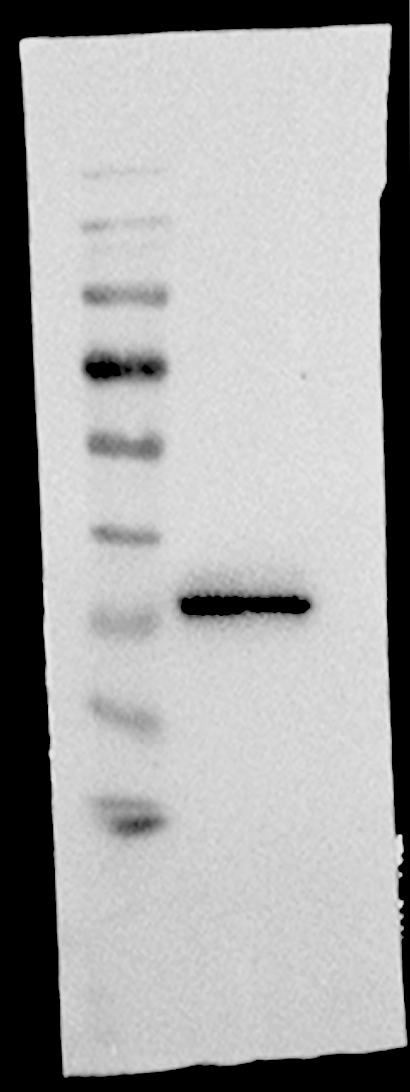

Supplement: Figure 6—figure supplement 2—source data 2. [file elife-97189-fig6-figsupp2-data2.zip › Figure 6–figure supplement 2-Source Data 2/GFP.tif]

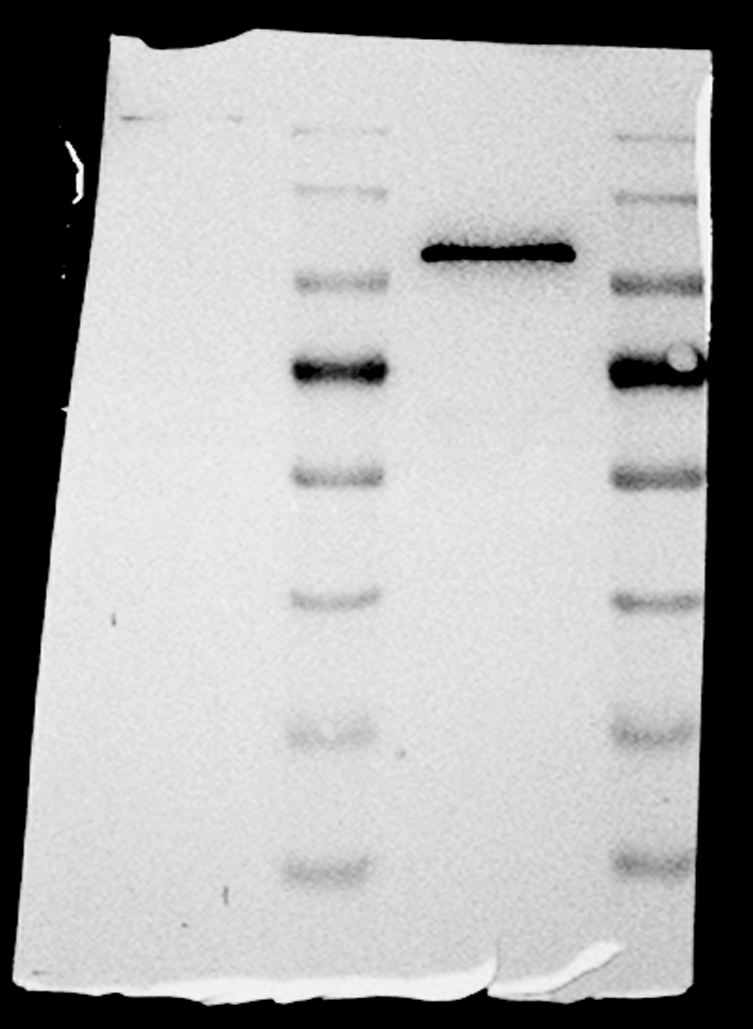

Supplement: Figure 6—figure supplement 2—source data 2. [file elife-97189-fig6-figsupp2-data2.zip › Figure 6–figure supplement 2-Source Data 2/NRK-GFP.tif]
