# Supplementary material for: Receptor tyrosine kinases CAD96CA and FGFR1 function as the cell membrane receptors of insect juvenile hormone
Source: eLife. 2025 Mar 14;13:RP97189. doi: 10.7554/eLife.97189 (PMC11908783; doi:10.7554/eLife.97189)
Supplement: Figure 3—figure supplement 1—source data 2. [file elife-97189-fig3-figsupp1-data2.pdf]

## Figure 3–figure supplement 1B

**MET1-CopGFP-His**

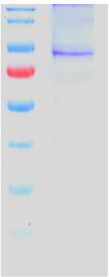

**CopGFP-His**

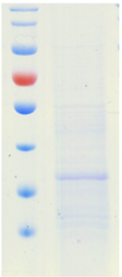

## Figure 3–figure supplement 1F

**CAD96CA-M1-CopGFP-His**

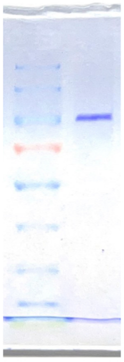

**CAD96CA-M2-CopGFP-His**

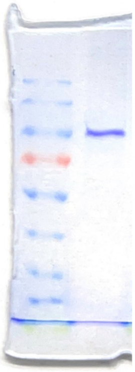

**CAD96CA-M3-CopGFP-His**

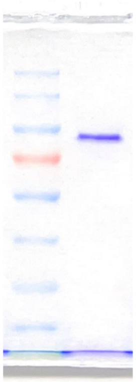

**CAD96CA-M4-CopGFP-His**

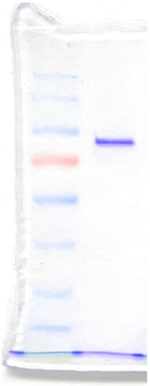

## Figure 3–figure supplement 1I

**FGFR1-M1-CopGFP-His**

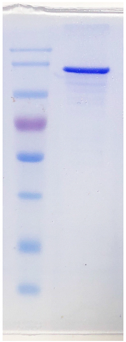

**FGFR1-M2-CopGFP-His**

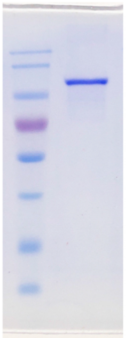

**FGFR1-M3-CopGFP-His**

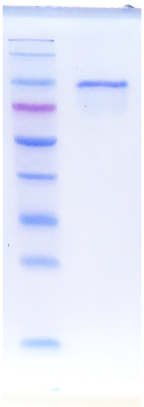

**FGFR1-M4-CopGFP-His**

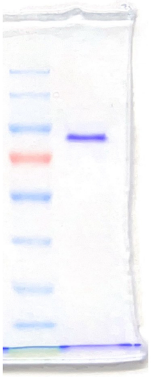

**Figure 3–figure supplement 1, Source Data 2. Original gel images corresponding to Figure 3–figure supplement 1B, F, and I.**
