# Supplementary material for: Receptor tyrosine kinases CAD96CA and FGFR1 function as the cell membrane receptors of insect juvenile hormone
Source: eLife. 2025 Mar 14;13:RP97189. doi: 10.7554/eLife.97189 (PMC11908783; doi:10.7554/eLife.97189)
Supplement: Figure 6—figure supplement 2—source data 1. [file elife-97189-fig6-figsupp2-data1.pdf]

Figure 6–figure supplement 2A

GFP

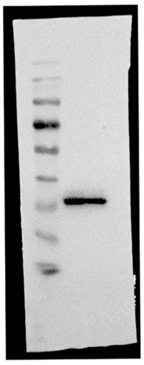

CAD96CA-GFP

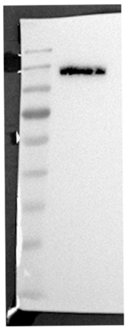

FGFR1-GFP

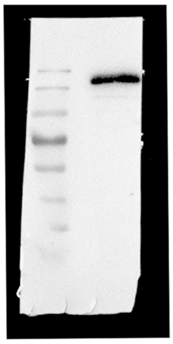

NRK-GFP

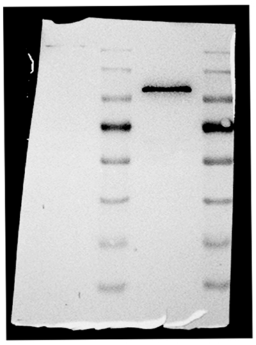

Figure 6–figure supplement 2B

CAD96CA-GFP

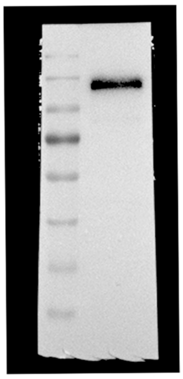

CAD96CA-M1-GFP

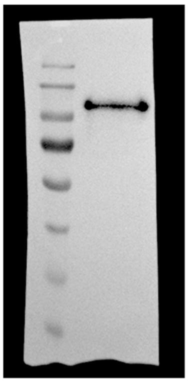

CAD96CA-M2-GFP

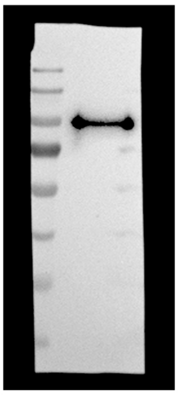

CAD96CA-M3-GFP

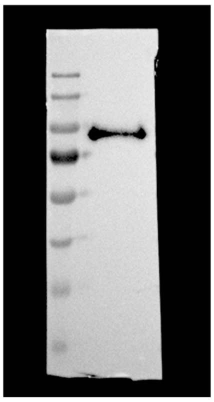

CAD96CA-M4-GFP

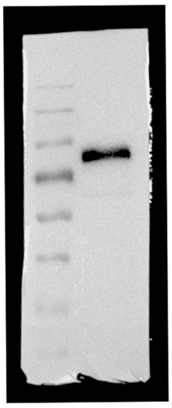

FGFR1-GFP

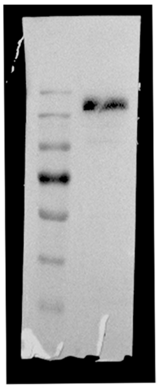

FGFR1-M1-GFP

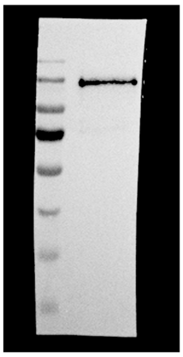

FGFR1-M2-GFP

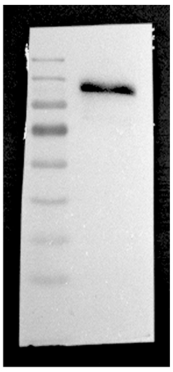

FGFR1-M3-GFP

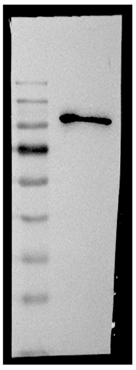

FGFR1-M4-GFP

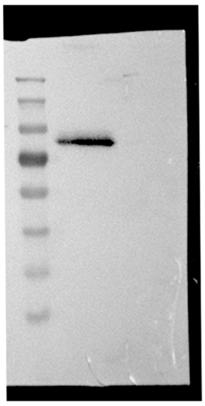

Figure 6–figure supplement 2, Source Data 1. Original western blot images corresponding to Figure 6–figure supplement 2A and B.
