# Supplementary material for: Receptor tyrosine kinases CAD96CA and FGFR1 function as the cell membrane receptors of insect juvenile hormone
Source: eLife. 2025 Mar 14;13:RP97189. doi: 10.7554/eLife.97189 (PMC11908783; doi:10.7554/eLife.97189)
Supplement: Supplementary file 1. [file elife-97189-supp1.doc]

Supplementary file 1. Names of RTKs identified in *H. armigera* genome

| Helicoverpa armigera | | Bombyx mori | Drosophila melanogaster |
| --- | --- | --- | --- |
| Name | Symol | Symol | Symol |
| ALK/Anaplastic lymphoma kinase | LOC110383585 | ALK | ALK |
| Cad96Ca/Cadherin 96Ca | LOC110379194 | Cad96Ca | Cad96Ca |
| Ddr/Discoidin domain receptor | LOC110378887 | TKP | Ddr |
| Dnt/Doughnut on | LOC110383864 | Dnt isoform X1 | Dnt |
| Drl/Derailed | LOC110383805 | Dnt | Drl |
| EDdr/Epithelial discoidin domain receptor | LOC110374488 | EDdr |  |
| EGFR/Epidermal growth factor receptor | LOC110375773 | EGFR | Egfr |
| EphB2/Ephrin type-B receptor 2 | LOC110379128 | EphB1 | EphB2 |
| FGFR1/Fibroblast growth factor receptor homolog 1 | LOC110373728 | FGFR | Htl/DFR1/Dtk1 |
| IGFR1/Insulin-like growth factor 1 receptor | LOC110381988 | LOC101741863 |  |
| InR/Insulin-like receptor | LOC110377777 | InR | InR |
| Nrk/Neurotropic receptor kinase | LOC110384207 | HOP | Nrk |
| Otk/Offtrack | LOC110377855 | Otk | Otk |
| Ror/Receptor tyrosine kinase orphan receptor | LOC110384348 | Ror | Ror |
| Ror-like isoform X1/  Receptor tyrosine kinase like orphan receptor | LOC110371076 | Ror isoform X1 | Ror |
| ROS/Proto-oncogene tyrosine-protein kinase | LOC110381275 | ROS | Sev |
| STE20-like/serine/  threonine-protein kinase STE20-like | LOC110370444 |  |  |
| Torso/tyrosine-protein kinase receptor torso like | LOC110371197 | Torso | Torso |
| VEGFR1/Vascular endothelial growth factor receptor 1 | LOC110383235 | VEGFR1 | Pvr |
| Wsck/Cell wall integrity and stress response component kinase | LOC110377380 | Wsck | Wsck |
