# Supplementary material for: Receptor tyrosine kinases CAD96CA and FGFR1 function as the cell membrane receptors of insect juvenile hormone
Source: eLife. 2025 Mar 14;13:RP97189. doi: 10.7554/eLife.97189 (PMC11908783; doi:10.7554/eLife.97189)
Supplement: Supplementary file 2. [file elife-97189-supp2.docx]

Supplementary file 1. Oligonucleotide sequences of PCR primers.

| Primer name | 5´ 3´ nucleotide sequence |
| --- | --- |
| **qRT-PCR** |  |
| Kr-h1-RTF | atgtttacgagatttcggttac |
| Kr-h1-RTR | atgtgggcttccatttgtttt |
| Jhi-1-RTF | accacatcttcatcacaacca |
| Jhi-1-RTR | tacaactcatccaagccctca |
| Jhi-26-RTF | gcggatacgaaccacat |
| Jhi-26-RTR | ggctccactgacacgat |
| Vg-RTF | gtcaatgaggatgaacaggga |
| Vg-RTR | gttggcgttagacacgagagg |
| Torso-RTF | cgggcagataagcacaactc |
| Torso-RTR | gaggaaaggctcgtttgatg |
| Otk-RTF | gtgcgtgattcgttcgtt |
| Otk-RTR | ccttctactcgacttgtggg |
| Ddr-RTF | gtgtccgaggtcgcaaat |
| Ddr-RTR | cgataacatacgcctctgc |
| Wsck-RTF | gattggagtggtggcagtt |
| Wsck-RTR | tgtggttgccaagggtat |
| Egfr-RTF | gactatctgatgccctcaccgc |
| Egfr-RTR | aaccgcaaatcctttattccct |
| Ste20-like-RTF | ctcgccacgctactccaca |
| Ste20-like -RTR | tcatactccgccgacagg |
| Vegfr1-RTF | ttaggttgaaagattacccacg |
| Vegfr1-RTR | atctccagtacgctcgtgtc |
| Ror-like-RTF | tcacgcacgaatcagacg |
| Ror-like-RTR | tggcggcacaagcacta |
| Fgfr1-RTF | gtggcaacggcgtgtctt |
| Fgfr1-RTR | aactctgctcttctgcgtatca |
| Ros-RTF | tcccgctcgtgagtatga |
| Ros-RTR | tgattgagtgttccgtgctat |
| Igfr1-RTF | tgctgctgtgcctgctggtg |
| Igfr1-RTR | cggtgccgagtttccgatta |
| Inr-RTF | tcttggtacaccgtgaacatc |
| Inr-RTR | actacgaagccgttggggttctgag |
| Dnt-RTF | cgagaaactaaggctgaaggtg |
| Dnt-RTR | gccagaggtgatgctccaag |
| Drl-RTF | agatgcgagggagcaagaagt |
| Drl-RTR | gctaacacccaggaccgacag |
| Cad96ca-RTF | ttcaacctacccgccatca |
| Cad96ca-RTR | tctccaacccataagtcacag |
| Alk-RTF | aagaaggcggtgatagacgatt |
| Alk-RTR | tgactgttggacgaggaggac |
| Nrk-RTF | ggactacagccaagtaaccac |
| Nrk-RTR | gaggtcttgtatgctgatgagggta |
| Ror-RTF | acacgccgcaaaggagac |
| Ror-RTR | ccgttggaagaggagcag |
| Ephb2-RTF | cagtgctggagacaaccttcg |
| Ephb2-RTR | tcggctgtttcttatcacattca |
| Eddr-RTF | atgcgacctgtcaccttccttg |
| Eddr-RTR | tgccgctttcacttcgttatgg |
| **RNAi** |  |
| Fgfr1-RNAiF | gcgtaatacgactcactatagggagcgtcactgaacgagag |
| Fgfr1-RNAiR | gcgtaatacgactcactatagggaaacgtggagggaaatat |
| Vegfr1-RNAiF | gcgtaatacgactcactatagggttgcctcacttcagcc |
| Vegfr1-RNAiR | gcgtaatacgactcactatagggtttcgcactttccacg |
| Wsck-RNAiF | gcgtaatacgactcactataggg tttctgtgggaatgcg |
| Wsck-RNAiR | gcgtaatacgactcactatagggggctggggtctggagt |
| Drl-RNAiF | gcgtaatacgactcactataggggagtggacttgccttgtacg |
| Drl-RNAiR | gcgtaatacgactcactatagggtcagctctgctatcctttgt |
| Cad96ca-RNAiF | gcgtaatacgactcactataggggtctacgccacagtctccga |
| Cad96ca-RNAiR | gcgtaatacgactcactatagggcgtctttcttgctatccttc |
| Ror-RNAiF | gcgtaatacgactcactatagggggcgtgtatttattgttt |
| Ror-RNAiR | gcgtaatacgactcactatagggggtgccattagtcttatc |
| Ephb2-RNAiF | gcgtaatacgactcactatagggatacccactggctcctgt |
| Ephb2-RNAiR | gcgtaatacgactcactatagggcattctcggcgtaaactt |
| Nrk-RNAiF | gcgtaatacgactcactatagggttatcgtgcttcttctta |
| Nrk-RNAiR | gcgtaatacgactcactatagggatgttgtggttacttggc |
| Ste20-like-RNAiF | gcgtaatacgactcactataggggcagaaaagacctacacagc |
| Ste20-like-RNAiR | gcgtaatacgactcactatagggcaggcaagtaacgtcacaac |
| **Overexpression** |  |
| Nrk-oveF | gattctagagctagcgaattcgccaccatggacattcactttaa |
| Nrk-oveR | tcgtcgctctccatagcggccgcttcaggatgagttctttccaatatca |
| Otk-oveF | gattctagagctagcgaattcgccaccatggtgatgtgcgtgattcgttcgttc |
| Otk-oveR | tcgtcgctctccatagcggccgcctcttcgactttctcctgagatttc |
| Cad96ca-oveF | gattctagagctagcgaattcgccaccatggtgatgtttctgacaagc |
| Cad96ca-oveR | tcgtcgctctccatagcggccgctagtttttctccatccaagtgctg |
| Fgfr1-oveF | gattctagagctagcgaattcgccaccatgaatctcgccg |
| Fgfr1-oveR | tcgtcgctctccatagcggccgctttgatgaaaggaaagtcactgtca |
| **Mutant** |  |
| Cad96ca-M1-F | gattctagagctagcgaattcgccaccatggtgagggtgtaccgtgaag |
| Cad96ca-M1-R | tcgtcgctctccatagcggccgctagtttttctccatccaagtgctg |
| Cad96ca-M2-F | gattctagagctagcgaattcgccaccatggtgtgggtgacagcatacg |
| Cad96ca-M2-R | tcgtcgctctccatagcggccgctagtttttctccatccaagtgctg |
| Cad96ca-M3-F | gattctagagctagcgaattcgccaccatggtgaggacgactcaaagca |
| Cad96ca-M3-R | tcgtcgctctccatagcggccgctagtttttctccatccaagtgctg |
| Cad96ca-M4-F | gattctagagctagcgaattcgccaccatggtgacagaagctcctaata |
| Cad96ca-M4-R | tcgtcgctctccatagcggccgctagtttttctccatccaagtgctg |
| Fgfr1-M1-F | gattctagagctagcgaattcgccacctgtaagactgataat |
| Fgfr1-M1-R  Fgfr1-M2-F  Fgfr1-M2-R | tcgtcgctctccatagcggccgctttgatgaaaggaaagtcactgtca  gattctagagctagcgaattcgccacccaccctacaaaacttt  tcgtcgctctccatagcggccgctttgatgaaaggaaagtcactgtca |
| Fgfr1-M3-F | gattctagagctagcgaattcgccaccgctgaaaacttgaccg |
| Fgfr1-M3-R | tcgtcgctctccatagcggccgctttgatgaaaggaaagtcactgtca |
| Fgfr1-M4-F | gattctagagctagcgaattcgccaccggatacttgactgtat |
| Fgfr1-M4-R | tcgtcgctctccatagcggccgctttgatgaaaggaaagtcactgtca |
| Crispr-Cas9 mutant |  |
| Universal primer | aaaagcaccgactcggtgccactttttcaagttgataacggactagccttattttaacttgctatttctagctctaaaac |
| Cad96ca-gRNA1 | taatacgactcactataggaagggtaatgttggtggggttttagagctagaa |
| Cad96ca-gRNA2 | taatacgactcactatagggtatcatcaggaggattgttttagagctagaa |
| Cad96ca-gRNAF1 | aagtggaagggtaatgttggtggggt |
| Cad96ca-gRNAR1 | taaaaccccaccaacattacccttcc |
| Cad96ca-gRNAF2 | aagtgggtatcatcaggaggattgt |
| Cad96ca-gRNAR2 | taaaacaatcctcctgatgataccc |
| Cad96ca-testF | gacagaagtctacgccaca |
| Cad96ca-testR  Fgfr1-gRNA1  Fgfr1-gRNA2  Fgfr1-gRNAF1  Fgfr1-gRNAR1  Fgfr1-gRNAF2  Fgfr1-gRNAR2  Fgfr1-testF  Fgfr1-testR  gRNAwf-F  gRNAwf-R  Met1-gRNA  Met1-testF  Met1-testR | gcatacaaacaggatcaca  taatacgactcactatagggaggctgcgactgacctggttttagagctagaa  taatacgactcactataggagcagagttgtgcagcaggttttagagctagaa  aagtgagaggctgcgactgacctggt  taaaaccaggtcagtcgcagcctctc  aagtagagcagagttgtgcagcaggt  taaaacctgctgcacaactctgctct  acccaataaacaacctca  ctggtccttctactatacttac  tgattacgaattcccgggaggttatgtagtacacattg  gtgttttacgcgcccgggaaaaaaagcaccgactcggt  taatacgactcactatagaggggccccgaggcctattgttttagagctagaa  atgacatcttcaggcggag  ctatatcggacaacaaaga |
| **HEK-239T**  Overexpression  Cad96ca-W-F  Cad96ca-W-R  Cad96ca-M1-F  Cad96ca-M1-R  Cad96ca-M2-F  Cad96ca-M2-R  Cad96ca-M3-F  Cad96ca-M3-R  Cad96ca-M4-F  Cad96ca-M4-R  Fgfr1-W-F  Fgfr1-W-R  Fgfr1-M1-F  Fgfr1-M1-R  Fgfr1-M2-F  Fgfr1-M2-R  Fgfr1-M3-F  Fgfr1-M3-R  Fgfr1-M4-F  Fgfr1-M4-R  ***S. frugiperda***  **qRT-PCR**  Cad96ca-RTF  Cad96ca-RTR  Fgfr-RTF  Fgfr-RTR  Kr-h1-RTF  Kr-h1-RTR  **RNAi**  Cad96ca-RNAiF  Cad96ca-RNAiR  Fgfr-RNAiF  Fgfr-RNAiR  ***D. melanogaster***  **qRT-PCR**  Cad96ca-RTF  Cad96ca-RTR  Htl-RTF  Htl-RTR  Btl-RTF  Btl-RTR  Kr-h1-RTF  Kr-h1-RTR  **RNAi**  Cad96ca-RNAiF  Cad96ca-RNAiR  Htl-RNAiF  Htl-RNAiR  Btl-RNAiF  Btl-RNAiR | ctcgagaccatggtggaattcatgtttctgacaagcgtctggg  ctcgcccttgctcatggtacctagtttttctccatccaagtgctg  ctcgagaccatggtggaattcagggtgtaccgtgaaggcagt  ctcgcccttgctcatggtacctagtttttctccatccaagtgctg  ctcgagaccatggtggaattctgggtgacagcatacgacggc  ctcgcccttgctcatggtacctagtttttctccatccaagtgctg  ctcgagaccatggtggaattcaggacgactcaaagcactacc  ctcgcccttgctcatggtacctagtttttctccatccaagtgctg  ctcgagaccatggtggaattcacagaagctcctaataagaat  ctcgcccttgctcatggtacctagtttttctccatccaagtgctg  ctcgagaccatggtggaattcatgaatctcgccgccattg  ctcgcccttgctcatggtacctttgatgaaaggaaagtcactgtca ctcgagaccatggtggaattctgtaagactgataatgataatg  ctcgcccttgctcatggtacctttgatgaaaggaaagtcactgtca ctcgagaccatggtggaattccaccctacaaaactttacaaaat  ctcgcccttgctcatggtacctttgatgaaaggaaagtcactgtca ctcgagaccatggtggaattcgctgaaaacttgaccgttgtag  ctcgcccttgctcatggtacctttgatgaaaggaaagtcactgtca ctcgagaccatggtggaattcggatacttgactgtattggaat  ctcgcccttgctcatggtacctttgatgaaaggaaagtcactgtca  tgaacttgaacttccctgcct  tcaccacgagaactcctatgc  tggctccagagtcgctttat  ccaggtcttccaccagttca  tctgtcttcgtgatttcggtta  gcctccatttgtttttttgtgt  gcgtaatacgactcactataggtgcctaccccagtttaccta  gcgtaatacgactcactataggccgttgtcccaagactcata  gcgtaatacgactcactataggcgaaggaggatgaagggtg  gcgtaatacgactcactataggcgactggcattagcaaagc  tcaaggaaagtgccaccgaagt  gcagcccaacaaatgaacaaca  tcaaggaaagtgccaccgaagt  gcagcccaacaaatgaacaaca  tagtggctttgttccttttgg  acggttgttctctgcttctcg  cctaccactgtgacatctgctt  tccatctccttcttgttcttga  gcgtaatacgactcactatagggggagagcagagtggagaa  gcgtaatacgactcactataggcagggaacatagcgattgg  gcgtaatacgactcactatagggggagagcagagtggagaa  gcgtaatacgactcactataggcagggaacatagcgattgg  gcgtaatacgactcactataggggctacccttttgcgactt  gcgtaatacgactcactataggcaccgtgtgctttctgctt |
